# ZFPM2-AS1 facilitates cell growth in esophageal squamous cell carcinoma via up-regulating TRAF4

**DOI:** 10.1042/BSR20194352

**Published:** 2020-04-03

**Authors:** Gaozhong Sun, Changhao Wu

**Affiliations:** Department of Cardio-Thoracic Surgery, Zhejiang Provincial People’s Hospital, People’s Hospital of Hangzhou Medical College, Hangzhou 310014, Zhejiang Province, P.R. China

**Keywords:** esophageal squamous cell carcinoma, miR-3612, TRAF4, ZFPM2-AS1

## Abstract

Emerging evidence has confirmed that long noncoding RNAs (lncRNAs) are strongly involved in tumor initiation and development. LncRNA ZFPM2 antisense RNA 1 (ZFPM2-AS1) has been identified as a tumor facilitator in some cancers; nevertheless, its functional significance and regulatory mechanism remain greatly unclear in esophageal squamous cell carcinoma (ESCC). Here, we detected ZFPM2-AS1 expression in ESCC cell lines using qRT-PCR. ZFPM2-AS1 knockdown models were established for investigating the biological function of ZFPM2-AS1 in ESCC cells. The association between miR-3612 and ZFPM2-AS1 or TRAF4 was assessed by RNA pull-down and luciferase reporter assays. The present study indicated that ZFPM2-AS1 was significantly up-regulated in ESCC cells. Functional assays manifested that ZFPM2-AS1 knockdown restrained cell proliferation, migration and invasion, and facilitated cell apoptosis in ESCC. Mechanistically, ZFPM2-AS1 promoted ESCC cell growth and up-regulated TRAF4 to trigger NF-κB pathway by sequestering miR-3612. Besides, miR-3612 was confirmed to be a tumor inhibitor in ESCC. Through restoration experiments, we observed that TRAF4 overexpression could recover the suppressive effect of ZFPM2-AS1 on ESCC cell growth. Collectively, all the results suggested that ZFPM2-AS1 was an oncogene in ESCC cell growth by up-regulating TRAF4 and activating NF-κB pathway.

## Introduction

Esophageal cancer, a commonly occurred cancer, causes the increasing deaths that related to cancers worldwide [1]. Esophageal squamous cell carcinoma (ESCC) is the predominant histological type of esophageal cancer and accounts for most esophageal cancer cases [2]. Hot drinks, consumption of pickled vegetables, excessive drinking and mycotoxin-contaminated foods are all identified as risk factors that contribute to esophageal cancer [3]. In recent decades, great advances have been used in ESCC treatment; however, the clinical outcomes are still unfavorable [4]. In consequence, it is of great importance to explore the novel biomarkers and mechanisms underlying ESCC progression for identifying attractive therapeutic strategies.

Long noncoding RNAs (lncRNAs), consisting of over 200 nucleotides, are identified as a group of RNA with a limit in encoding proteins [5]. As reported, lncRNA functions as a regulator in gene expression via multiple mechanisms, such as chromatin modifications, miRNA competition, protein amounts and genomic interactions [6,7]. Existing evidence proved that lncRNAs play crucial roles in the regulation of various biological and pathological behaviors, especially in tumorigenesis and progression [8,9]. For example, lncRNA ZEB1-AS1 acts as an oncogene in glioma and promotes cancer progression [10]. LncRNA HULC promotes cell proliferation and invasion and activates PI3K/AKT pathway in pancreatic cancer [11]. Notably, a series of abnormally expressed lncRNAs are found in ESCC, and these lncRNAs are reported to be strongly associated with ESCC malignancy [12]. For instance, SNHG6 [13] and LINC01980 [14] are up-regulated in ESCC and function as tumor promoter in ESCC progression. Contrarily, GAS5 [15] and FER1L4 [16] are down-regulated in ESCC and suppress the cancer progression. LncRNA ZFPM2 antisense RNA 1 (ZFPM2-AS1) was reported to play oncogenic role in gastric cancer [17] and lung adenocarcinoma [18]. However, it expression pattern and biological function have not been elucidated in ESCC.

Here, we explored the function and molecular mechanism of ZFPM2-AS1 in ESCC, and found that ZFPM2-AS1 was expressed at a high level in ESCC and promoted ESCC cell growth by targeting miR-3612/TRAF4 axis. The present study might provide a useful theoretical basis for the understanding of potential regulatory mechanism underlying ESCC progression.

## Materials and methods

### Cell culture

Human ESCC cell lines (KYSE-140, KYSE-30, EC9706, TE-10), and human normal esophageal epithelial cell line (HET-1A), from ATCC (Rockville, Maryland), were propagated in the DMEM (Invitrogen, Carlsbad, CA) under the standard condition of 37°C and 5% CO_2_. The 10% FBS and 1% Pen/Strep mixture, both from Invitrogen, served as supplements for DMEM.

### Quantitative real-time PCR (qRT-PCR)

The total RNAs were extracted the cultured cell samples with Invitrogen TRIzol reagent, then prepared for cDNA synthesis with reverse transcription kit (Thermo Fisher, Waltham, MA). Gene expression levels were monitored by qPCR with SYBR green Supermix (Thermo Fisher), then calculated with the comparative change-in-cycle method (ΔΔCt), relative to that of GAPDH or U6.

### Transfection

The designed short hairpin RNAs (shRNAs) and control shRNA were acquired from Genepharma Company (Shanghai, China) to silence ZFPM2-AS1 with transfection kit Lipofectamine 2000 (Invitrogen). Besides, the miRNA mimics and NC mimics, along with pcDNA-TRAF4 and control pcDNA3.1 vector, all were also obtained from Genepharma Company. At 48 h post-transfection, cell samples were reaped.

### EdU assay

EC9706 and TE-10 cells were transfected as study designed, then seeded on sterile coverslips in the 24-well plates. EdU assay kit from Ribobio (Guangzhou, China) was employed for detecting proliferative cells as per the protocol. Nuclei were double stained with EdU and DAPI dye, followed by analysis with fluorescence microscope (Olympus, Tokyo, Japan).

### TUNEL assay

Transfected cells in 2% formaldehyde were reaped and premeabilized on ice with 0.1% Triton X-100. Samples were then incubated under 37°C with TUNEL reaction buffer as required by protocol of TUNEL assay kit (Beyotime, Shanghai, China). One hour later, fluorescence microscope was used.

### Wound-healing

For the scratch wound-healing assay, cell samples were seeded in serum-free medium for 24 h, and then wounded by pipette tips. The culture medium was then refreshed. Distance of wound healing was monitored at 0 and 24 h, images were taken.

### Transwell invasion assay

Matrigel-coated transwell chamber was available from Corning Co. (Corning, NY) for cell invasion assay. Cell samples were seeded in the serum-free culture medium before plating in the upper chamber, while complete culture medium was added to the lower chamber. Invasive cell samples on the bottom were fixed by 4% paraformaldehyde 24 h later, and treated with Crystal Violet dye, then observed under microscope.

### FISH

The specific FISH-probe of ZFPM2-AS1 was synthesized by Ribobio. Fixed cell samples were rinsed in PBS and dehydrated, then cultured with probe in the hybridization solution overnight at 42°C. At least, DAPI staining and fluorescence analysis were severally conducted.

### RNA immunoprecipitation (RIP)

Cell extracts were collected from RIP lysis buffer for incubation for 4 h in RIP buffer adding the magnetic beads coated human Ago2 antibody or control IgG antibody (Abcam, Cambridge, MA). After elution, precipitated RNAs were extracted and analyzed.

### Luciferase reporter assay

293T cells (ATCC) were used to co-transfected with the pmirGLO dual-luciferase vector (Promega Corporation, Madison, WI) containing the ZFPM2-AS1 sequences and various miRNA mimics and NC mimics. Besides, the wild-type and mutated ZFPM2-AS1 or TRAF4 fragments covering the miR-3612 binding sites were inserted to pmirGLO vector, termed ZFPM2-AS1-WT/MUT and TRAF4-WT/MUT. They were co-transfected into ESCC cell samples with miR-3612 mimics or NC mimics for 48 h, then assayed via luciferase reporter assay system (Promega).

### RNA pull down assay

The wild-type and mutated miR-3612 sequences covering ZFPM2-AS1 or TRAF4 binding sites were acquired and biotin-labeled into Biotin-miR-3612 WT/MUT. Then they were mixed with the protein extracts from ESCC cell samples and magnetic beads. The retrieved RNA enrichment was assayed.

### Statistical analyses

Averaged replicates of three independent assays were required in the present study, and data were displayed with mean ± Standard Deviation (S.D.). PRISM 6 (GraphPad, San Diego, CA) was utilized for statistical analyses by one-way ANOVA and Student’s *t*-test, with significant values specified as *P* < 0.05.

## Results

### ZFPM2-AS1 is overexpressed in ESCC and promotes ESCC cell growth

**Figure 1:**
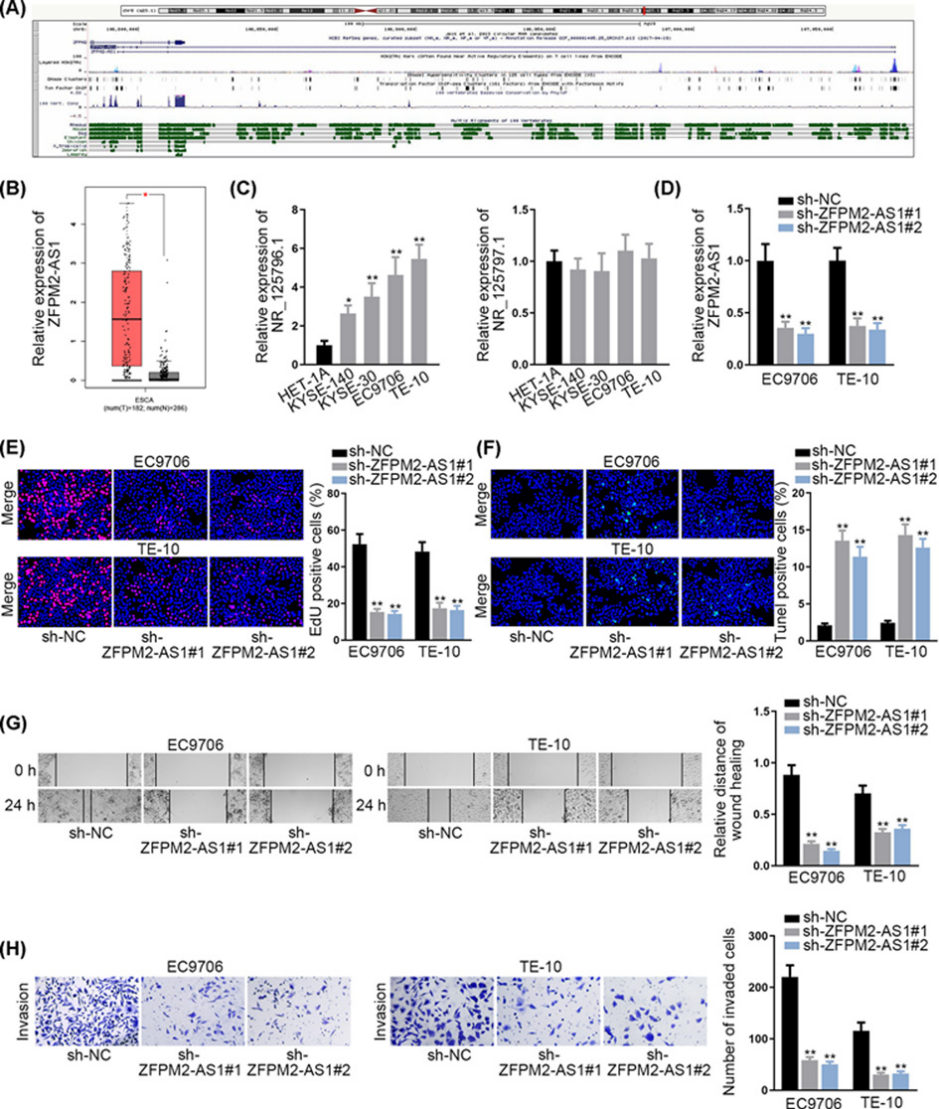
ZFPM2-AS1 is overexpressed in ESCC and promotes ESCC cell growth (**A**) ZFPM2-AS1 in UCSC database. (**B**) ZFPM2-AS1 expression in ESCC tissues through GEPIA. (**C**) Expressions of two transcripts of ZFPM2-AS1 in ESCC cell lines (KYSE-140, KYSE-30, EC9706 and TE-10) and human normal esophageal epithelial cell line (HET-1A). (**D**) Transfection efficiency of sh-ZFPM2-AS1 (for NR_125796.1). (**E**) Cell proliferation was detected by EdU assay upon ZFPM2-AS1 knockdown. (**F**) TUNEL assay was used for assessing the apoptosis in ZFPM2-AS1 silenced cells. (**G** and **H**) Cell migration and invasion in sh-ZFPM2-AS1 transfected cells were evaluated by wound healing and transwell assays; *P <0.05, **P <0.01.

To explore the expression pattern of ZFPM2-AS1 in ESCC, we first searched UCSC (http://genome.ucsc.edu/) database, and two transcripts were found in the genome of ZFPM2-AS1 (Figure 1A). Later, it was discovered that ZFPM2-AS1 was expressed at a high level in esophageal carcinoma tissues (Figure 1B). Further, we detected the expressions of two transcripts (NR_125796.1 and NR_125797.1) of ZFPM2-AS1 in ESCC cell lines (KYSE-140, KYSE-30, EC9706 and TE-10), and human normal esophageal epithelial cell line (HET-1A) was taken as a reference. The results showed that NR_125796.1 was remarkably up-regulated in ESCC cell lines, particularly in EC9706 and TE-10 cells; whereas NR_125797.1 presented no significant difference (Figure 1C). Therefore, NR_125796.1 in ZFPM2-AS1 genome was used for the follow-up studies. To explore the function of ZFPM2-AS1 on ESCC cell growth, two specific ZFPM2-AS1 shRNAs (sh-ZFPM2-AS1#1 and sh-ZFPM2-AS1#2, both for NR_125796.1) were used for stably silencing ZFPM2-AS1 expression in EC9706 and TE-10 cells (Figure 1D). Through EdU assay, we observed that the proliferative capacity of EC9706 and TE-10 cells was markedly restrained upon ZFPM2-AS1 knockdown (Figure 1E). In TUNEL assay, the depletion of ZFPM2-AS1 substantially promoted the apoptosis in EC9706 and TE-10 cells (Figure 1F). Furthermore, wound healing assay manifested that cell migration was obviously inhibited in sh-ZFPM2-AS1 transfected cells (Figure 1G). Subsequently, the invaded cells in sh-ZFPM2-AS1 group were validated to be fewer than in sh-NC group via transwell assay (Figure 1H). Besides, we knocked NR_125797.1 down and identified that silenced NR_125797.1 had no effects on ESCC cell proliferation, apoptosis, migration and invasion (Supplementary Figure S1A–C). These findings suggested that ZFPM2-AS1 (NR_125796.1 but not NR_125797.1) is overexpressed in ESCC and promotes ESCC cell growth.

### MiR-3612 is sponged by ZFPM2-AS1 and serves as a tumor suppressor in ESCC

**Figure 2:**
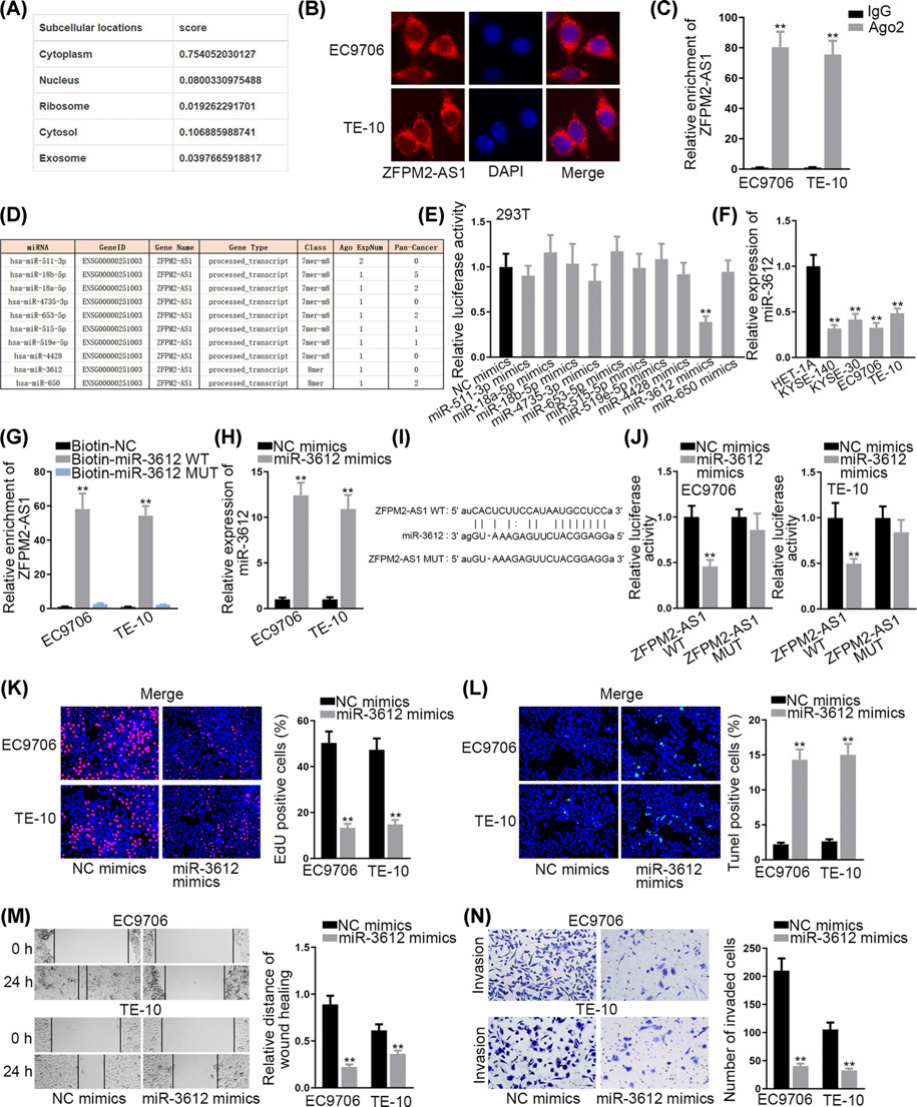
MiR-3612 is sponged by ZFPM2-AS1 and serves as a tumor suppressor in ESCC (**A**) The subcellular distribution of ZFPM2-AS1 from lncLocator database. (**B**) FISH assay was conducted to determine ZFPM2-AS1 localization. (**C**) RIP assay was performed for confirming the involvement of ZFPM2-AS1 in RISC. (**D**) Predicted miRNAs for ZFPM2-AS1. (**E**) Luciferase reporter assay was carried out to validate the interaction between ZFPM2-AS1 and predicted miRNAs. (**F**) MiR-3612 expression in ESCC cells and HET-1A cell. (**G**) RNA pull down assay was employed for detecting the combination between ZFPM2-AS1 and miR-3612. (**H**) MiR-3612 expression was detected in cells transfected with miR-3612 mimics. (**I**) Binding site between ZFPM2-AS1 and miR-3612 was showed. (**J**) Luciferase activity of ZFPM2-AS1-WT or ZFPM2-AS1-MUT was estimated in ESCC cells with the transfection of miR-3612 mimics or NC mimics. (**K**) Effect of miR-3612 mimics on cell proliferation. (**L**) Cell apoptosis was assessed by TUNEL assay after the transfection of miR-3612 mimics. (**M** and **N**) Wound healing and transwell assays were utilized for determining the effect of miR-3612 overexpression on cell migration and invasion; **P <0.01.

A variety of reports have pointed out that lncRNAs could sponge miRNAs by acting as competitive endogenous RNAs (ceRNAs) at post-transcriptional level [19]. To elucidate potential ZFPM2-AS1 mechanism, lncLocator (http://www.csbio.sjtu.edu.cn/bioinf/lncLocator/) [20] was searched and we found that ZFPM2-AS1 was primarily located in the cytoplasm (Figure 2A), which was consistent with the results of subcellular fractionation assay (Figure 2B). This result suggested that ZFPM2-AS1 might exert its function as a ceRNA. To test our hypothesis, RIP assay was carried out and results indicated that ZFPM2-AS1 could enrich in the beads conjugated with Ago2 antibody (Figure 2C), providing the evidence for ceRNA hypothesis. Using starBase (http://starbase.sysu.edu.cn/index.php) [21], 10 potential miRNAs sponged by ZFPM2-AS1 were predicted (Figure 2D). Among these miRNAs, miR-3612 was found to significantly suppress the luciferase activity of the vectors containing ZFPM2-AS1 sequence (Figure 2E). Therefore, miR-3612 was chosen for the following exploration. Then, miR-3612 expression level in ESCC cells and normal esophageal epithelial cell line (HET-1A) was detected. Compared with HET-1A cell line, miR-3612 was lowly expressed in ESCC cells (Figure 2F). RNA pull-down assay showed that ZFPM2-AS1 was pulled down by biotinylated miR-3612-WT (Figure 2G). In addition, we overexpressed miR-3612 in EC9706 and TE-10 cells (Figure 2H). The binding site between miR-3612 and ZFPM2-AS1 was illustrated in Figure 2I. Results of luciferase reporter assay displayed that the overexpression of miR-3612 markedly lessened the luciferase activity of ZFPM2-AS1-WT reporter in EC9706 and TE-10 cells while such effect was not exerted on ZFPM2-AS1-Mut reporter (Figure 2J). Subsequently, we investigated the biological effect of miR-3612 on ESCC cell growth. It was observed that miR-3612 overexpression suppressed the proliferation of EC9706 and TE-10 cells (Figure 2K). The apoptosis of ESCC cells was enhanced with the transfection of miR-3612 mimics (Figure 2L). Furthermore, the migratory and invasive capacities were both hampered in miR-3612 overexpressed ESCC cells (Figure 2M,N). Taken all together, miR-3612 is sponged by ZFPM2-AS1 and serves as a tumor suppressor in ESCC.

### TRAF4 is a target gene of miR-3612

**Figure 3:**
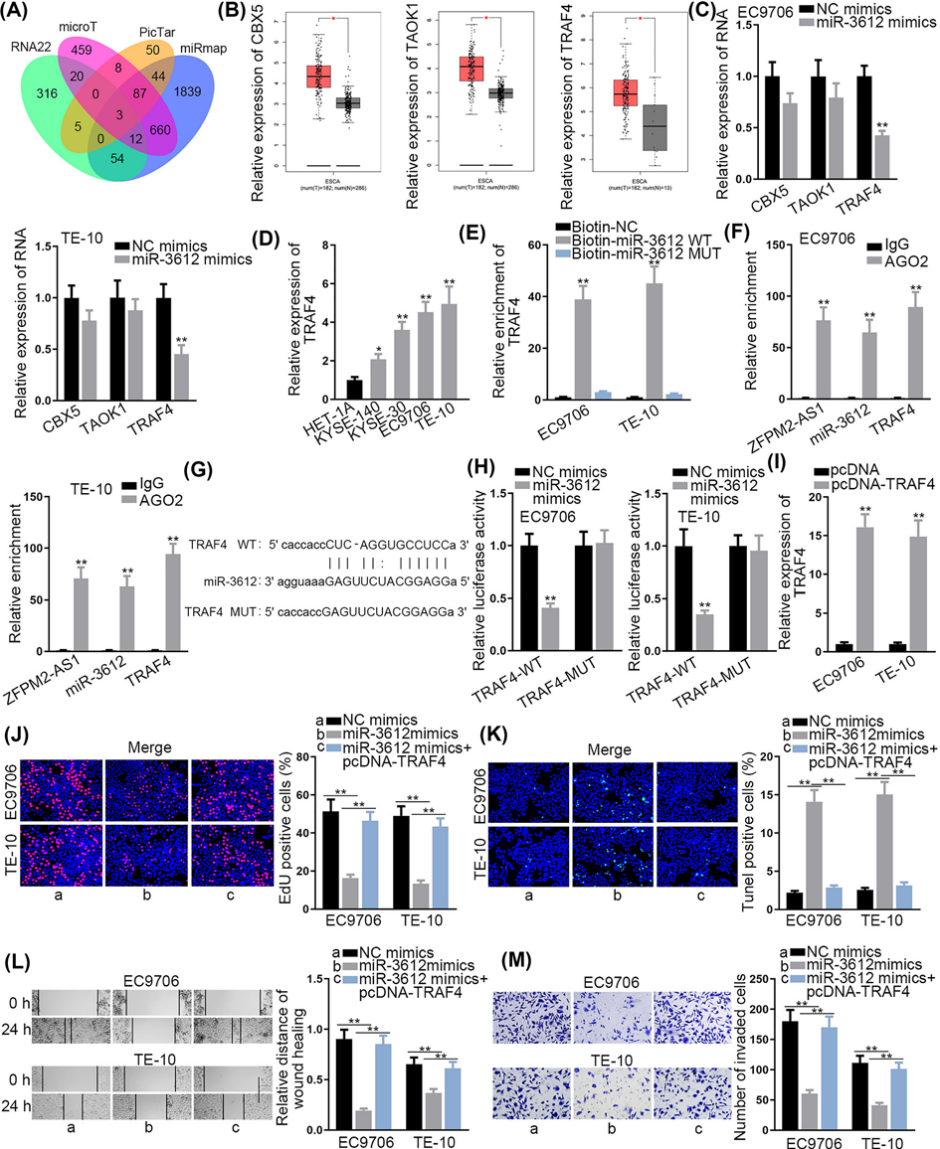
TRAF4 is a target gene of miR-3612 (**A**) Predicted mRNAs were showed by Venn diagram. (**B**) CBX5, TAOK1 and TRAF4 expressions in ESCC tissues. (**C**) The expressions of CBX5, TAOK1 and TRAF4 in ESCC cells transfected with miR-3612 mimics. (**D**) TRAF4 expression in ESCC cells and HET-1A cell. (**E**) The potential interaction between TRAF4 and miR-3612 was validated by RNA pull-down assay. (**F**) RIP assay was conducted for measuring the binding between TRAF4 and miR-3612. (**G**) Predicted binding site of miR-3612 on TRAF4. (**H**) Luciferase activity of TRAF4-WT or TRAF4-MUT was estimated in ESCC cells with the transfection of miR-3612 mimics or NC mimics. (**I**) Transfection efficiency of pcDNA-TRAF4 in ESCC cells was detected. (**J** and **K**) Cell proliferation and apoptosis were determined by transfecting miR-3612 mimics**.** (**L** and **M**) Function of up-regulated miR-3612 on cell migration and invasion; *P <0.05, **P <0.01.

To further support ceRNA hypothesis, we explored the target gene of miR-3612. Online target exploratory software tools (RNA22, microT, PicTar and miRmap) were utilized to search the potential targets for miR-3612, and 3 candidates were screened out (Figure 3A). Through GEPIA database (http://gepia.cancer-pku.cn/), we found that these 3 mRNAs (CBX5, TAOK1 and TRAF4) were all up-regulated in esophageal carcinoma tissues (Figure 3B). Then, their expression levels were detected in the cells transfected with miR-3612 mimics. The results displayed that miR-3612 overexpression decreased the expression of TRAF4, while exerted no effect on that of CBX5 and TAOK1 (Figure 3C). Therefore, TRAF4 was predicted as a target of miR-3612. Later, it was discovered that TRAF4 was highly expressed in ESCC cells (Figure 3D). Furthermore, RNA pull-down assay confirmed the potential interaction of miR-3612 with TRAF4 (Figure 3E). RIP assay depicted the significant enrichment of ZFPM2-AS1, miR-3612 and TRAF4 in the complex precipitated by Ago2 antibody (Figure 3F). As Figure 3G presented, miR-3612 was predicted to have a putative binding site on TRAF4. We also discovered that the luciferase activity of TRAF4-WT reporter was diminished with the transfection of miR-3612 mimics, while that of TRAF4-Mut reporter was not affected (Figure 3H). To further confirm the interaction between miR-3612 and TRAF4, TRAF4 expression was elevated for the rescue assays (Figure 3I). EdU assay delineated that the repressed cell proliferation in miR-3612 up-regulated cells was facilitated by TRAF4 overexpression (Figure 3J). Additionally, the up-regulation of miR-3612 boosted cell apoptosis, while the transfection of pcDNA-TRAF4 recovered this effect (Figure 3K). Finally, TRAF4 overexpression offset the function of miR-3612 up-regulation on cell migration and invasion (Figure 3L,M). Since TRAF4 had sigificant effects on NF-κB pathway, we then detected protein expression of p65 and phospho-p65 (p-p65) via Western blotting analysis. The results demonstrated that p-p65 expression was reduced by silenced ZFPM2-AS1 but was recovered again by up-regulated TRAF4 (Supplementary Figure S2A). Collectively, TRAF4 was a target gene of miR-3612. ZFPM2-AS1 sequestered miR-3612 to up-regulate TRAF4, activating NF-κB pathway.

### ZFPM2-AS1 enhances ESCC cell growth via up-regulating TRAF4

**Figure 4:**
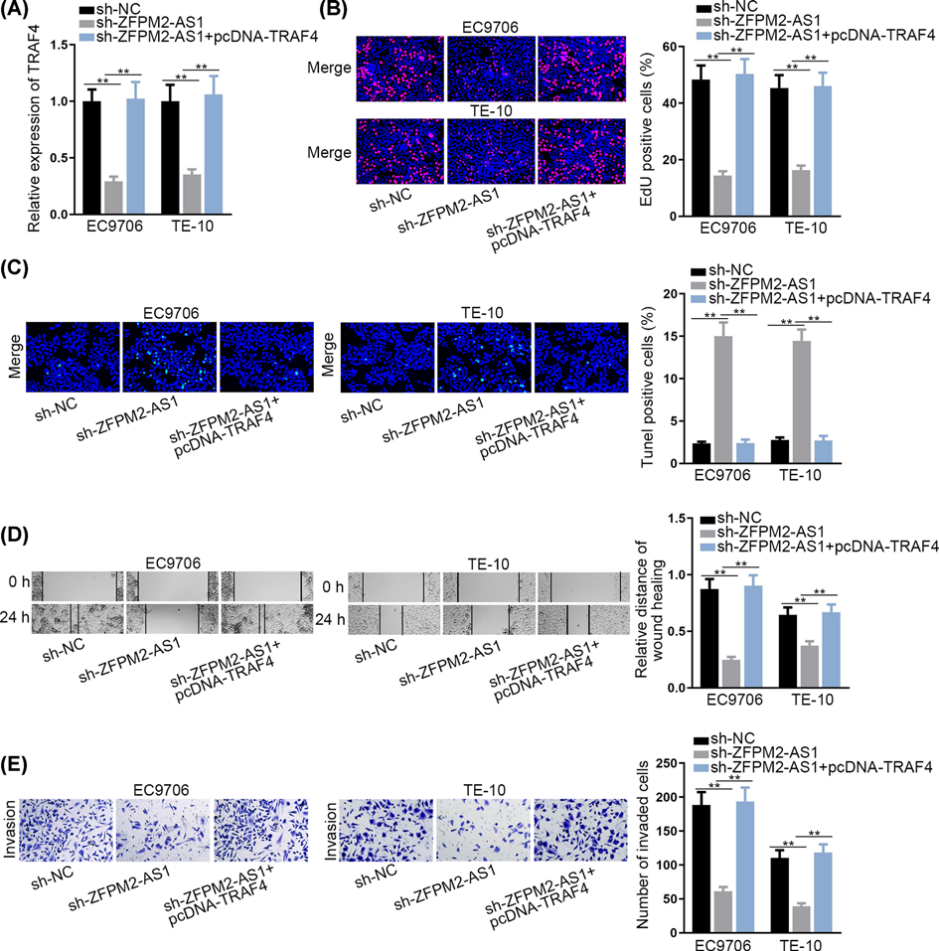
ZFPM2-AS1 enhances ESCC cell growth via up-regulating TRAF4 (**A**) TRAF4 expression was detected in cells transfected with sh-NC, sh-ZFPM2-AS1, sh-ZFPM2-AS1+pcDNA-TRAF4. (**B** and**C**) EdU and TUNEL assays were performed to evaluate cell proliferation and apoptosis in each group. (**D** and**E**) The migration and invasion in each group was confirmed by wound healing and transwell assay; **P <0.01.

For the purpose of probing whether ZFPM2-AS1 promoted ESCC cell growth by regulating TRAF4 expression, some restoration experiments were designed and carried out. The results of qRT-PCR implied that the decreased TRAF4 expression in sh-ZFPM2-AS1 transfected cells was reserved by transfecting pcDNA-TRAF4 (Figure 4A). EdU assay implied that the suppressed cell proliferation by ZFPM2-AS1 knockdown was abrogated by TRAF4 up-regulation (Figure 4B). In TUNEL assay, the promotive effect of sh-ZFPM2-AS1 on the apoptosis was recovered by up-regulated TRAF4 (Figure 4C). Wound healing assay and transwell assay unveiled that cell migration and invasion hampered by ZFPM2-AS1 silencing was abolished by overexpressed TRAF4 (Figure 4D,E). In conclusion, ZFPM2-AS1 enhances ESCC cell growth via up-regulating TRAF4.

## Discussion

Accumulating studies have uncovered the aberrant expression of lncRNAs in ESCC [22]. The dysregulated lncRNAs were validated to be closely correlated with ESCC malignancy through affecting tumor processes [23]. Thus, it may be essential to investigate the functions of lncRNAs in ESCC progression for identifying novel potential biomarkers for the therapy of cancers. Here, we first detected ZFPM2-AS1 expression in ESCC cells and then evaluated its effect on the cell growth, including proliferation, apoptosis, migration and invasion. ZFPM2-AS1 acted as a tumor promoter in tumorigenesis and tumor progression. For example, ZFPM2-AS1 enhances cell proliferation, inhibits cell apoptosis and alleviates the p53 pathway in gastric cancer [17]. ZFPM2-AS1 is overexpressed in lung adenocarcinoma, predicts a poor prognosis and boosts cell proliferation [18]. In the present study, we found that ZFPM2-AS1 was up-regulated in ESCC cells. The knockdown of it was associated with the inhibited cell proliferation, migration, invasion and augmented apoptosis. These results uncovered the oncogenic property of ZFPM2-AS1 in ESCC.

Diverse mechanisms by which ZFPM2-AS1 modulates cancer progression have been well revealed, including stabilizing MIF in gastric cancer [17] and sponging miR-18b-5p to regulate VMA21 expression in lung adenocarcinoma [18]. Notably, the potential molecular mechanisms by which ZFPM2-AS1 might modulate the tumorigenic processes in ESCC were explored. MicroRNAs (miRNAs) are small RNAs comprising 20–22 nucleotides [24]. MiRNAs could emerge as significant regulators in tumor initiation and progression [25]. Extensive reports have unveiled a series of dysregulated miRNAs in ESCC [26,27]. MiR-3612 is a novel miRNA that has not been studied in cancers. In the present study, we found that miR-3612 was expressed at a low level in ESCC cells. Besides, miR-3612 could interact with ZFPM2-AS1. Furthermore, the overexpression of miR-3612 hampered cell proliferation, migration and invasion and facilitated cell apoptosis in ESCC. Conclusively, ZFPM2-AS1 acted as miR-3612 sponge.

It has been confirmed that miRNAs could recognize and directly bind to their target mRNAs 3′ untranslated regions (3′-UTRs) and consequently leading to the degradation or translation inhibition of mRNAs [28]. TRAF4, TNF receptor associated factor 4, is targeted by miR-302c-3p and overexpressed in hepatocellular carcinoma [29]. In addition, TRAF4 acts as functional target of miR-29a/b/c in glioma and its expression level was strongly correlated with the prognosis of patients [30]. In the present study, we discovered that TRAF4 was highly expressed in ESCC cells and served as a target gene of miR-3612. Additionally, overexpressed TRAF4 recovered the effect of miR-3612 or ZFPM2-AS1 up-regulation on ESCC cell growth.

It has been widely reported that lncRNAs are potential crucial regulators of inflammatory signaling, including NF-κB pathway [31]. NF-κB is a significant contributor to initiation and progression of various cancers via controlling tumor cell proliferation, survival, migration, inflammation, invasion and angiogenesis [32]. Also, NF-κB pathway was reported to facilitate ESCC cell proliferation and invasion [33]. The previous studies have pointed out that TRAF4 could activate NF-κB to accelerate cancer development [34,35]. In present study, we identified that ZFPM2-AS1 could activate NF-κB pathway via up-regulating TRAF4, thus facilitating ESCC cell proliferation, migration, invasion and inhibiting ESCC cell apoptosis.

In conclusion, ZFPM2-AS1 acted as an up-regulated lncRNA in ESCC cells and enhanced ESCC cell growth by sequestering miR-3612 to up-regulate TRAF4 and activate NF-κB pathway. Thus, ZFPM2-AS1/miR-3612/TRAF4 might provide a theoretical basis for treating ESCC.

## Supplementary Material

Supplementary Figures S1-S2

## Abbreviations

ceRNA: competitive endogenous RNA
ESCC: esophageal squamous cell carcinoma
lncRNA: long noncoding RNA
RIP: RNA immunoprecipitation
shRNA: short hairpin RNA
