# Supplementary material for: ZFPM2-AS1 facilitates cell growth in esophageal squamous cell carcinoma via up-regulating TRAF4
Source: Biosci Rep. 2020 Apr 3;40(4):BSR20194352. doi: 10.1042/BSR20194352 (PMC7133517; doi:10.1042/BSR20194352)
Supplement: Supplementary Figures S1-S2 [file BSR-2019-4352_supp.pdf]

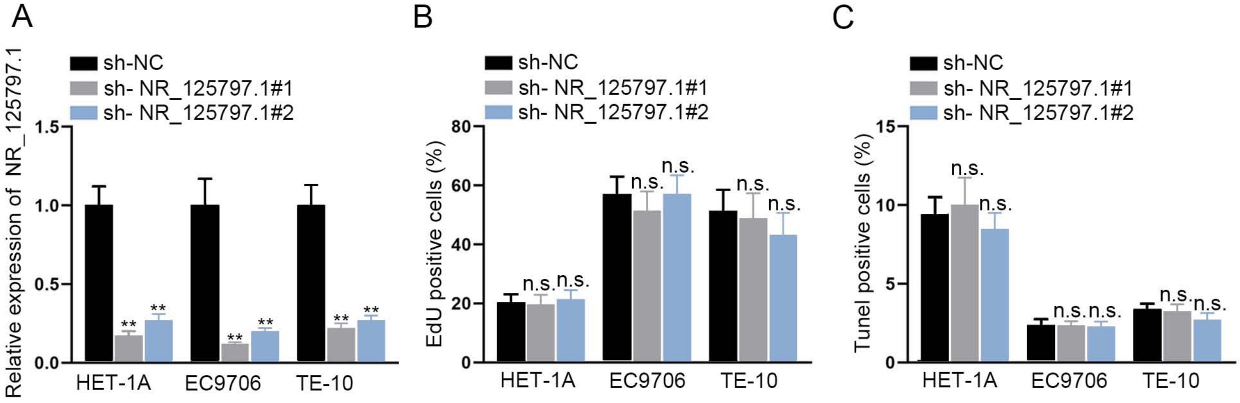

A
B
C
sh-NC
sh- NR_125797.1#1
sh- NR_125797.1#2
Relative expression of NR_125797.1
EdU positive cells (%)
Tunel positive cells (%)
1.5
1.0
0.5
0.0
80
60
40
20
0
15
10
5
0
**
n.s.
HET-1A
EC9706
TE-10

Figure S1 (A) Depletion efficiency of NR_125797.1 was validated through qRT-PCR assay. (B-C) EdU and TUNEL assays evaluated cell proliferation and apoptosis by depletion of NR_125797.1. $^{**}P < 0.01$. “n.s.” indicates no significance.

A

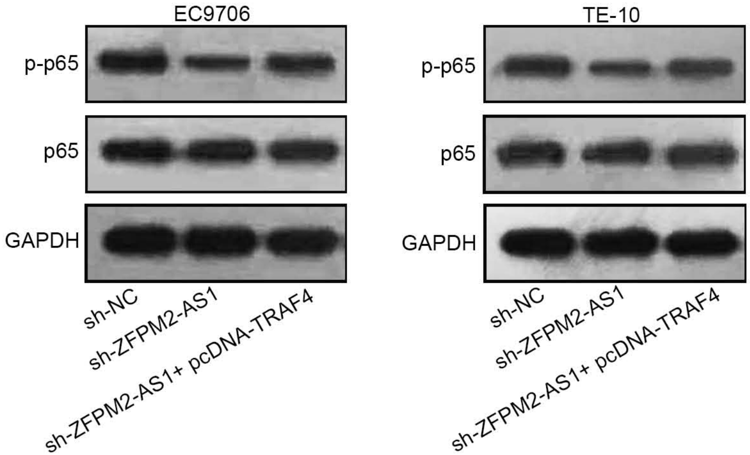
EC9706
TE-10
p-p65
p65
GAPDH
sh-NC
sh-ZFPM2-AS1
sh-ZFPM2-AS1+ pcDNA-TRAF4

Figure S2 (A) Western blotting revealed p65 and p-p65 protein levels in 3 groups: sh-NC, sh-ZFPM2-AS1, sh-ZFPM2-AS1+pcDNA-TRAF4.
